# Pharmacokinetic Study of Thirteen Ingredients after the Oral Administration of *Flos Chrysanthemi* Extract in Rats by UPLC-MS/MS

**DOI:** 10.1155/2020/8420409

**Published:** 2020-08-21

**Authors:** Qi Jia, Xuhua Huang, Guangzhe Yao, Wenjuan Ma, Jiayuan Shen, Yanxu Chang, Huizi Ouyang, Jun He

**Affiliations:** ^1^First Teaching Hospital of Tianjin University of Traditional Chinese Medicine, Tianjin 300193, China; ^2^Tianjin State Key Laboratory of Modern Chinese Medicine, Tianjin University of Traditional Chinese Medicine, Tianjin 301617, China

## Abstract

A rapid and reliable UPLC-MS/MS method was developed and validated for the simultaneous quantification of thirteen bioactive compounds (luteolin, cynaroside, luteolin 7-O-glucuronide, isochlorogenic acid C, chlorogenic acid, cryptochlorogenic acid, apigenin, apigenin 7-glucoside, acacetin, hyperoside, isoquercitrin, tilianin, and hesperidin) in rat plasma. The compounds were separated on an ACQUITY UPLC BEH C_18_ column (2.1 × 100 mm, 1.7 *μ*m) with a gradient mobile phase system of acetonitrile and 0.1% (*v*/*v*) formic acid aqueous solution at a flow rate of 0.3 mL/min. All compounds were quantitated using Agilent Jet Stream electrospray ionization (AJS ESI) in a negative ion mode. The lower limit of quantification (LLOQ) for all compounds was below 5 ng/mL. The intra- and interday accuracy ranged from -13.0% to 14.0%, and precisions were less than 12.2%. The extraction recoveries of the compounds were in the range of 56.9% to 95.0%, and the matrix effect ranged between 71.6% and 109.3%. Stability studies proved that the thirteen compounds were stable under tested conditions, with a relative standard deviation (RSD) of less than 11.4%. This developed method was successfully applied to the pharmacokinetic study of the 13 bioactive compounds after oral administration of *Flos Chrysanthemi* extract in rat by UPLC-MS/MS. Pharmacokinetic parameters of 8 out of the 13 compounds investigated are presented in this paper.

## 1. Introduction

*Flos Chrysanthemi* is a dried capitulum belonging to the Compositae family and is native to Asia and northeastern Europe [1–3]. There are 7 different varieties of *Flos Chrysanthemi*, namely, Haoju, Chuju, Gongju, Hangju (Dabaiju, Huju, and Xiaobaiju), and Huaiju [4]. As a traditional Chinese medicine and popular herbal tea in China, *Flos Chrysanthemi* is commonly used to dispel wind, reduce heat, calm a hyperactive liver, and improve eyesight [4, 5]. Various studies have identified three different types of bioactive compounds in *Flos Chrysanthemi*, namely, flavonoids, caffeic acid, and phenolics, which provide a series of pharmacological actions such as antioxidative activity, anti-inflammatory activity, anticancer activity, lipid-lowering effect in a fatty liver, and antiangiogenic activity [6–13].

Pharmacokinetics plays an important role in drug development by quantitatively describing various dynamic processes in the body. At present, there are a few pharmacokinetic studies on luteolin, apigenin, diosmetin, and chrysoeriol in oral *Flos Chrysanthemi* extract [14–16]. The pharmacokinetics of *Flos Chrysanthemi* has not been comprehensively evaluated in the existing studies because its extract contains a wide variety of chemical compounds. Hence, it is of relevance to further research the pharmacokinetics of various ingredients in rat plasma after the oral intake of *Flos Chrysanthemi* extract.

This research develops a sensitive and reliable UPLC-MS/MS method for the determination of thirteen compounds in rat plasma after oral administration of *Flos Chrysanthemi* extract. In addition, this is the first pharmacokinetic study on the chemical components luteolin 7-O-glucuronide and apigenin 7-glucoside. This study would provide reference for further pharmacological studies on *Flos Chrysanthemi* extract.

## 2. Experimental

### 2.1. Chemicals, Reagents, and Materials

Acetonitrile (Thermo Fisher Scientific, Fair Lawn, NJ, USA), methanol (Thermo Fisher Scientific, Fair Lawn, NJ, USA), and formic acid (ROE SCIENTIFIC INC, Newark, USA, MO, USA) were of HPLC grade. Water used in the experiment was purified with Milli-Q advanced ultrapure water system (Millipore, Milford, MA, USA). Luteolin, cynaroside, luteolin 7-O-glucuronide, isochlorogenic acid C, chlorogenic acid, cryptochlorogenic acid, apigenin, apigenin 7-glucoside, acacetin, hyperoside, isoquercitrin, tilianin, hesperidin, and icariin (internal standard (IS)) (purity ≥ 99%) were purchased from Chengdu Must Bio-Technology Co., Ltd. (Chengdu, China).

### 2.2. Apparatus and Analytical Conditions

**Figure 1:**
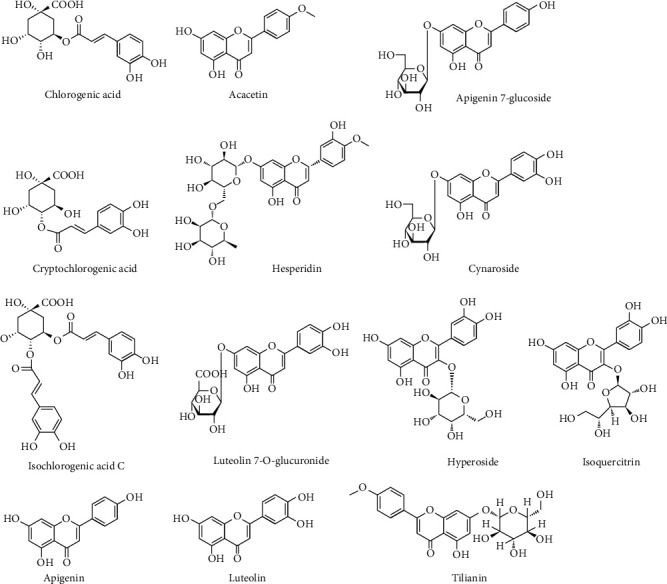
The chemical structures of the 13 ingredients.

The UPLC-MS/MS system was made up of Agilent 1290 UPLC combined with an Agilent 6470 series triple quadrupole mass spectrometer, and AJS ESI was selected as a source. The chromatographic separation was performed on an ACQUITY UPLC BEH C_18_ (2.1 × 100 mm, 1.7 *μ*m) column, set at 20°C. Mobile phases used were 0.1% (*v*/*v*) formic acid in water (A) and acetonitrile (B). The following elution gradient was used: 0-2 min, 2%-25% B; 2-4 min, 25%-26% B; 4-9 min, 26%-75% B; and 9-10 min, 75%-90% B. The flow rate was maintained at 0.3 mL/min, and a 5 *μ*L sample was injected each time. Quantitative parameters are displayed in Table 1. And the chemical structures of 13 ingredients are shown in Figure 1.

### 2.3. The Acquisition of *Flos Chrysanthemi* Extract

To prepare the *Flos Chrysanthemi* extract, 1000 g of *Flos Chrysanthemi* powder was weighed and extracted twice using 14 L of 70% (*v*/*v*) ethanol via reflux for 80 min each time, according to Chinese Pharmacopoeia (2015). The extracted solutions were then filtered, mixed, and evaporated under reduced pressure. The *Flos Chrysanthemi* extract was pressed into powder and stored in a dryer until analysis. The extract contained luteolin, cynaroside, luteolin 7-O-glucuronide, isochlorogenic acid C, chlorogenic acid, cryptochlorogenic acid, apigenin, apigenin 7-glucoside, acacetin, hyperoside, isoquercitrin, tilianin, and hesperidin at 14.00, 17.11, 1.63, 5.18, 3.10, 0.65, 0.70, 3.89, 0.01, 0.01, 0.01, 0.21, and 0.22 mg/g, respectively.

### 2.4. Stock and Working Solution

Stock solutions of luteolin, cynaroside, luteolin 7-O-glucuronide, isochlorogenic acid C, chlorogenic acid, cryptochlorogenic acid, apigenin, apigenin 7-glucoside, acacetin, hyperoside, isoquercitrin, tilianin, hesperidin, and icariin (internal standard solution) were prepared individually and diluted to 1 mg/mL with methanol. Appropriate amounts of the 13 different stock solutions were added together in methanol for the mixed standard solution. The calibration solutions were prepared by adding 20 *μ*L IS and appropriate volumes of mixed standard solution into 100 *μ*L blank rat plasma. Low, medium, and high concentrations of quality control (QC) samples consisting of appropriate mixed standard solutions and blank plasma sample, adjusted to desired concentrations, were selected as calibration solutions. These solutions were kept at 4°C.

### 2.5. Sample Preparation

20 *μ*L methanol (volume corresponding to that of QC samples and calibration curve) and 20 *μ*L IS (1 *μ*g/mL) were added into 100 *μ*L plasma sample, which was then vortex-mixed with 400 *μ*L acetonitrile for 3 min. The mixed solution was then centrifuged for 10 min at 14,000 rpm. After collecting the supernatant in a clean Eppendorf tube, the supernatant was evaporated to dryness under a gentle stream of nitrogen gas. The residue was dissolved in 100 *μ*L methanol, vortex-mixed for 3 min, and then centrifuged for 10 min at 14,000 rpm. Finally, 5 *μ*L supernatant was used for analysis by the UPLC-MS/MS system.

### 2.6. Method Validation

#### 2.6.1. Specificity

Plasma samples acquired at 0.08 h after oral administration of *Flos Chrysanthemi* extract were compared to spiked plasma samples (containing working solutions and IS) and blank plasma samples from six different rats to evaluate method specificity and identify endogenous interfering substances.

#### 2.6.2. The Calibration Curves and LLOQ

Blank rat plasma individually spiked with different concentrations of mixed standard solution and IS was quantitatively measured for three consecutive days, in replica, to validate the linearity. Calibration curves were drawn with peak-area ratios (*y*) of analyte relative to IS against its minimal concentration (*x*). The weighting factor was1/*x*^2^. The lowest limit of quantification (LLOQ) was calculated based on a signal-to-noise ratio of approximately 10 (S/N ≥ 10).

#### 2.6.3. Precision and Accuracy

Intra- and interday precision and accuracy were estimated by analysing six duplicated QC samples at different concentrations as follows: 1, 10, and 200 ng/mL for tilianin and hesperidin; 5, 50, and 1000 ng/mL for isochlorogenic acid C, chlorogenic acid, cryptochlorogenic acid, apigenin, apigenin 7-glucoside, acacetin, hyperoside, and isoquercitrin; 10, 100, and 2000 ng/mL for luteolin 7-O-glucuronide; and 20, 200, and 4000 ng/mL for luteolin and cynaroside. A standard calibration curve was plotted based on the above readings. Intraday and interday precisions were measured by RSD, and accuracy was measured by relative error (RE) of respective readings.

#### 2.6.4. The Recovery and Matrix Effect

Mixed working solution with IS, extracted samples of plasma samples spiked with mixed working solution and IS, and postextraction blank samples (containing IS) spiked with mixed working solution of three QC concentrations were quantitatively analysed in the same assay. Recovery was measured with peak-area differences between the corresponding extraction and postextraction spiked samples. A matrix effect was measured with peak-area differences between the corresponding extraction samples and mixed working solution. Six parallel experiments were performed in total.

#### 2.6.5. Stability

To study the stability of all compounds in rat plasma, low, medium, and high concentrations of QC samples were tested under the following test conditions: stored at an autosampler for 12 hours, stored at room temperature for 6 hours, put through three freeze/thaw cycles, and stored at -80°C for 14 days. Three QC concentrations were tested for each of the above conditions.

### 2.7. The Study of Pharmacokinetics

Six male Sprague-Dawley rats (250 ± 5 g) were purchased from Huafukang Bioscience Co., Inc. (Beijing, China). The rats were adapted to the facility for a week. Before the experiments, rats were fasted for 12 h, although drinking water was readily accessible. *Flos Chrysanthemi* extract was dissolved in CMC-Na aqueous solution and orally administered to the animals once at 10 g/kg. Blood samples (220 *μ*L) were taken from the orbital venous plexus of rats at 0, 0.03, 0.08, 0.17, 0.25, 0.5, 1, 2, 4, 6, 8, 10, 12, and 24 h after extract administration. Blood samples were promptly centrifuged at 6000 rpm for 10 min, and plasma samples were collected. The obtained plasma was stored at -80°C until analysis. “Drug and Statistics 3.0” software (Medical College of Wannan, China) was used to calculate pharmacokinetic parameters. The animal protocol was approved by the Animal Ethics Committee of Tianjin University of Traditional Chinese Medicine (TCM-LAEC20190056).

## 3. Results

### 3.1. LC-MS/MS Optimization

Various types of mobile phases were investigated for optimal separation of the 13 compounds, such as the use of acetonitrile or methanol as mobile (B) and 0.05% or 0.1% formic acid in water as mobile (A). Experimental results showed that acetonitrile (B) and 0.1% (*v*/*v*) formic acid in water (A) provided better peak shapes and reduced separation timings. Both positive and negative ion modes of the AJS ESI source were experimented for optimal mass spectrometry results. A negative ion mode showed greater signal intensity; thus, the thirteen compounds were quantitated with AJS ESI in the negative ion mode.

### 3.2. Sample Preparation

In the study, we identified two simple and efficient methods for processing plasma samples: protein precipitation and liquid-liquid extraction. The ethyl acetate liquid-liquid extraction method provided better recovery of flavonoids, but the recoveries of chlorogenic acid, cryptochlorogenic acid, and isochlorogenic acid C were poor. The effects of methanol and acetonitrile on protein precipitation were then compared, and the results demonstrated that acetonitrile precipitation produced better recovery rates.

### 3.3. Method Validation of Bioanalysis

#### 3.3.1. Specificity

**Figure 2:**
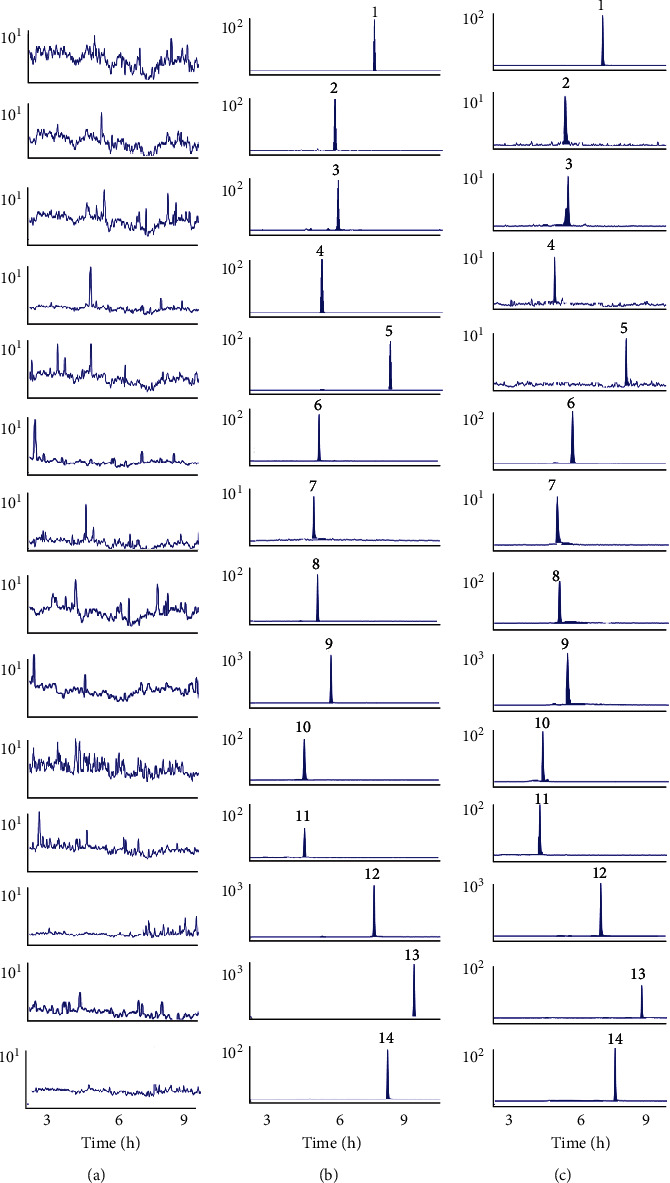
MRM chromatograms of hesperidin (1), isochlorogenic acid C (2), isoquercitrin (3), hyperoside (4), luteolin 7-O-glucuronide (5), cynaroside (6), tilianin (7), apigenin 7-glucoside (8), chlorogenic acid (9), cryptochlorogenic acid (10), luteolin (11), acacetin (12), apigenin (13), and IS (14). (a) Blank rat plasma chromatograms, (b) blank rat plasma samples added to chemical compounds and IS, and (c) plasma samples after oral *Flos Chrysanthemi* extract administration.

The respective chromatograms were compared to evaluate method specificity based on analyte retention times and the presence of interference peaks.  Figure 2 shows chromatograms of (a) blank plasma samples, (b) blank plasma samples spiked with analytes and IS, and (c) plasma sample collected after oral administration of *Flos Chrysanthemi* extract. Results showed consistent retention times and no interference peaks for all analytes across samples.

#### 3.3.2. Linearity and LLOQ

Values of LLOQs and calibration curves are displayed in Table 2. The range of calibration curves for tilianin and hesperidin was 1-200 ng/mL; for isochlorogenic acid C, cryptochlorogenic acid, chlorogenic acid, apigenin, acacetin, hyperoside, isoquercitrin, and apigenin 7-glucoside, it was 5-1000 ng/mL; for luteolin 7-O-glucuronide, it was 10-2000 ng/mL; and for luteolin and cynaroside, it was 20-4000 ng/mL. Results showed that the compounds exhibited good linearity in the linear range *r*^2^ > 0.9919. LLOQs were all below 5 ng/mL, indicating high sensitivity.

#### 3.3.3. Precision and Accuracy

The intraday and interday precision and accuracy were measured with six replicates of QC samples, with analytes set at low, medium, and high concentrations as described above. The results of the analysis are shown in Table 3. Accuracy (RE) ranged from -13.0% to 14.0%, and precision (RSD) ranged from 0.4% to 12.2%, indicating that the developed method was reliable.

#### 3.3.4. Recovery and Matrix Effect

As shown in Table 4, the extraction recovery of all 13 analytes, at three different concentrations, ranged between 56.9% and 95.0%. The matrix effects on all analytes ranged between 71.6% and 109.3%. These results indicated that both the matrix effect and extraction recovery are satisfactory.

#### 3.3.5. Stability

The stability of all 13 analytes during sample collection and processing was evaluated with the various storage conditions being tested on spiked plasma samples at three QC concentrations. As shown in Table 5, the RSD of all tested samples were below 11.4%, suggesting that all 13 analytes were stable in the above four test conditions.

### 3.4. Pharmacokinetic Study

**Figure 3:**
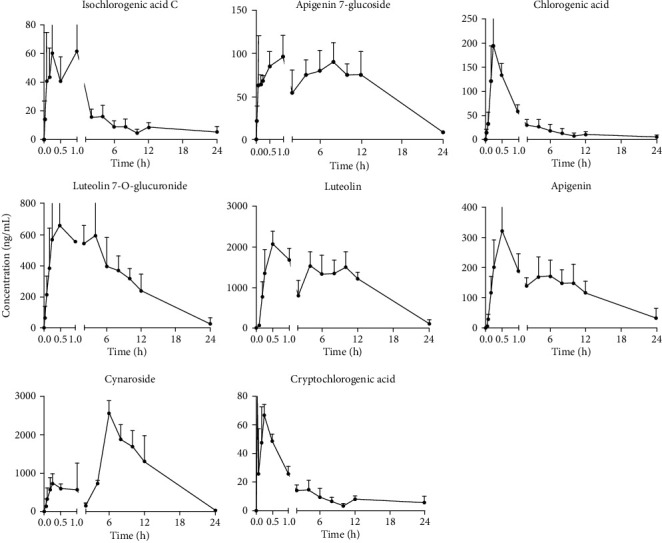
Primary concentration-time curves of isochlorogenic acid C, apigenin 7-glucoside, chlorogenic acid, luteolin 7-O-glucuronide, luteolin, apigenin, cynaroside, and cryptochlorogenic acid after oral administration of *Flos Chrysanthemi* extract (*n* = 6, mean ± SD).

Plasma samples from rats orally administered with *Flos Chrysanthemi* extract (10.0 g/kg) were analysed with UPLC-MS/MS. Plasma concentration-time curves are shown in Figure 3, and the primary pharmacokinetic parameters of each analyte are summarized in Table 6.

Five analytes, namely, acacetin, hyperoside, isoquercitrin, tilianin, and hesperidin, were detected only at the first few blood sampling points following oral administration of the *Flos Chrysanthemi* extract, which made it difficult to plot a complete pharmacokinetic curve. Hence, these 5 analytes were excluded in the following results.

As shown in Table 6, *C*_max_ of cynaroside and luteolin were 2547.84 ± 1121.18 ng/mL and 2079.55 ± 307.09 ng/mL, respectively, ranking as the highest two amongst the 8 remaining analytes. In addition, AUC_(0 − tn)_ of cynaroside and luteolin were larger than the other analytes, indicating a higher level of plasma exposure.

*T*_max_ of chlorogenic acid, cryptochlorogenic acid, cynaroside, luteolin 7-O-glucuronide, apigenin 7-glucoside, isochlorogenic acid C, apigenin, and luteolin were 0.25 ± 0.00 hours, 0.61 ± 0.33 hours, 6.38 ± 3.80 hours, 0.50 ± 0.27 hours, 0.69 ± 0.49 hours, 0.40 ± 0.31 hours, 0.50 ± 0.00 hours, and 0.50 ± 0.00 hours, respectively. Results showed that besides cynaroside, the other seven compounds were rapidly absorbed into the bloodstream. Meanwhile, *T*_max_ of apigenin and luteolin were similar to the values of previous reports [15].

*T*_1/2_ of chlorogenic acid and isochlorogenic acid C are 0.20 h and 0.24 h, respectively, indicating that these two compounds are eliminated shortly after oral administration. *T*_1/2_ of cynaroside, luteolin 7-O-glucuronide, cryptochlorogenic acid, apigenin 7-glucoside, apigenin, and luteolin range from 5.01 h to 13.87 h, suggesting that these compounds have a relatively longer therapeutic time, especially apigenin 7-glucoside.

## 4. Conclusion

A reliable and sensitive UPLC-MS/MS method was developed to measure 13 ingredients (luteolin, cynaroside, luteolin 7-O-glucuronide, isochlorogenic acid C, chlorogenic acid, cryptochlorogenic acid, apigenin 7-glucoside, apigenin, acacetin, hyperoside, isoquercitrin, tilianin, and hesperidin) after the oral administration of *Flos Chrysanthemi* extract in rat plasma. This method offered adequate specificity, precision, recovery, and stability. In addition, the results showed that the blood concentrations of cynaroside and luteolin were higher than the other 11 analytes following oral administration of the *Flos Chrysanthemi* extract. Meanwhile, the absorption and elimination of chlorogenic acid and isochlorogenic acid C were rapid compared to other compounds. These pharmacokinetic parameters facilitate further development and clinical application for *Flos Chrysanthemi.*

## Figures and Tables

**Table 1:** Mass spectra properties of 13 analytes and IS.

Compounds	Precursor ion (*m*/*z*)	Product ion (*m*/*z*)	Frag. (*V*)	C.E. (*V*)
Chlorogenic acid	353.1	191.0	98	20
Cryptochlorogenic acid	353.1	173.0	108	16
Cynaroside	449.1	286.9	113	24
Tilianin	447.1	285.0	128	20
Luteolin 7-O-glucuronide	461.1	285.0	141	24
Apigenin 7-glucoside	431.1	268.0	189	36
Isochlorogenic acid C	515.1	173.0	123	32
Luteolin	285.0	133.0	151	40
Apigenin	269.0	117.0	141	36
Hesperidin	609.2	301.0	146	28
Isoquercitrin	463.1	300.0	151	32
Hyperoside	463.1	270.9	156	52
Acacetin	283.1	268.0	128	24
Icariin (IS)	675.2	513.1	194	28

**Table 2:** Calibration curves, correlation coefficients, linear ranges, and LLOQ of the 13 analytes.

Compounds	Calibration curves	Correlation coefficients (*r*^2^)	Linear range (ng/mL)	LLOQ (ng/mL)
Luteolin	*Y* = 6.422005*X* + 0.176762	0.9964	20.0-4000.0	0.1
Cynaroside	*Y* = 0.047193*X* + 0.015018	0.9937	20.0-4000.0	0.1
Luteolin 7-O-glucuronide	*Y* = 6.507693*X* + 0.091156	0.9927	10.0-2000.0	2.0
Isochlorogenic acid C	*Y* = 2.288985*X* + 0.006645	0.9919	5.0-1000.0	5.0
Cryptochlorogenic acid	*Y* = 3.232775*X* − 0.004798	0.9990	5.0-1000.0	5.0
Chlorogenic acid	*Y* = 8.628364*X* + 0.060601	0.9943	5.0-1000.0	0.2
Apigenin	*Y* = 8.015200*X* + 0.032229	0.9993	5.0-1000.0	0.2
Acacetin	*Y* = 63.711025*X* + 1.046168	0.9984	5.0-1000.0	0.1
Hyperoside	*Y* = 7.003472*X* + 0.008604	0.9953	5.0-1000.0	0.2
Isoquercitrin	*Y* = 14.354529*X* + 0.013019	0.9958	5.0-1000.0	0.2
Apigenin 7-glucoside	*Y* = 20.395291*X* + 0.169449	0.9989	5.0-1000.0	0.1
Tilianin	*Y* = 53.090940*X* + 0.077200	0.9924	1.0-200.0	0.1
Hesperidin	*Y* = 4.330534*X* − 0.008981	0.9975	1.0-200.0	1.0

**Table 3:** Precision and accuracy of 13 analytes in rat plasma (*n* = 6).

Compounds	Spiked concentration (ng/mL)	Intraday			Interday		
		Measured concentration (ng/mL)	Accuracy (RE, %)	Precision (RSD, %)	Measured concentration (ng/mL)	Accuracy (RE, %)	Precision (RSD, %)
Luteolin	20	20.0 ± 0.4	0.0	2.0	20.4 ± 0.2	2.0	1.0
	200	187.6 ± 1.6	-6.2	0.9	188.5 ± 2.5	-5.8	1.3
	4000	4083.2 ± 46.1	2.1	1.1	4110.2 ± 65.5	2.8	1.6
Cynaroside	20	20.9 ± 0.9	4.5	4.3	20.5 ± 0.8	2.5	3.9
	200	208.1 ± 2.1	4.1	1.0	201.0 ± 0.9	0.5	0.4
	4000	3903.5 ± 58.1	-2.4	1.5	3921.2 ± 39.8	-2.0	1.0
Luteolin 7-O-glucuronide	10	9.0 ± 0.5	-10.0	5.6	8.9 ± 0.4	-10.0	4.5
	100	88.5 ± 0.6	-11.5	0.7	89.1 ± 2.1	-10.9	2.4
	2000	1840.1 ± 27.1	-8.0	1.5	1848.2 ± 36.6	-7.6	2.0
Isochlorogenic acid C	5	4.6 ± 0.2	-8.0	4.3	4.9 ± 0.3	-2.0	6.1
	50	44.4 ± 1.0	-11.2	2.3	45.1 ± 1.3	-9.8	2.9
	1000	1019.6 ± 26.6	2.0	2.6	1019.9 ± 34.6	2.0	3.4
Cryptochlorogenic acid	5	5.0 ± 0.2	0.0	4.0	5.0 ± 0.1	0.0	2.0
	50	44.0 ± 0.9	-12.0	2.0	44.2 ± 0.8	-11.6	1.8
	1000	1031.4 ± 24.1	3.1	2.3	1031.8 ± 25.0	3.2	2.4
Chlorogenic acid	5	4.9 ± 0.3	-2.0	6.1	5.0 ± 0.3	0.0	6.0
	50	46.7 ± 2.2	-6.6	4.7	47.3 ± 2.2	-5.4	4.7
	1000	1057.8 ± 21.6	5.8	2.0	1074.5 ± 27.7	7.5	2.6
Apigenin	5	5.6 ± 0.1	12.0	1.8	5.6 ± 0.1	12.0	1.8
	50	44.5 ± 1.2	-11.0	2.7	45.3 ± 1.2	-9.4	2.6
	1000	1043.9 ± 35.9	4.4	3.4	1048.8 ± 30.5	4.9	2.9
Acacetin	5	5.5 ± 0.2	10.0	3.6	4.9 ± 0.6	-2.0	12.2
	50	45.1 ± 0.7	-9.8	1.6	45.3 ± 1.1	-9.4	2.4
	1000	1038.1 ± 26.0	3.8	2.5	1044.8 ± 23.6	4.5	2.3
Hyperoside	5	4.6 ± 0.1	-8.0	2.2	4.7 ± 0.2	-6.0	4.3
	50	45.8 ± 1.1	-8.4	2.4	45.9 ± 1.3	-8.2	2.8
	1000	1019.7 ± 46.4	2.0	4.6	1032.2 ± 40.9	3.2	4.0
Isoquercitrin	5	4.8 ± 0.2	-4.0	4.2	4.9 ± 0.2	-2.0	4.1
	50	44.8 ± 0.5	-10.4	1.1	45.5 ± 1.1	-9.0	2.4
	1000	944.6 ± 41.8	-5.5	4.4	1000.7 ± 30.1	0.1	3.0
Apigenin 7-glucoside	5	5.7 ± 0.1	14.0	1.8	5.7 ± 0.1	14.0	1.8
	50	45.6 ± 1.0	-8.8	2.2	45.5 ± 1.0	-9.0	2.2
	1000	1078.0 ± 26.9	7.8	2.5	1070.0 ± 34.2	7.0	3.2
Tilianin	1	0.9 ± 0.1	-10.0	11.1	0.9 ± 0.1	-10.0	11.1
	10	9.7 ± 0.4	-3.0	4.1	9.8 ± 0.4	-2.0	4.1
	200	201.7 ± 2.5	0.8	1.2	200.7 ± 3.9	0.3	1.9
Hesperidin	1	1.1 ± 0.1	10.0	9.1	1.1 ± 0.1	10.0	9.1
	10	8.7 ± 0.2	-13.0	2.3	8.9 ± 0.2	-11.0	2.2
	200	223.6 ± 2.0	11.8	0.9	222.3 ± 2.0	11.2	0.9

**Table 4:** Extraction recoveries and matrix effects of the analytes (*n* = 6).

Compounds	Spiked concentration (ng/mL)	Extraction recovery (%)	RSD (%)	Matrix effect (%)	RSD (%)
Luteolin	20	91.0 ± 2.0	2.2	109.2 ± 3.6	3.3
	200	80.7 ± 10.5	13.0	96.6 ± 7.0	7.2
	4000	78.9 ± 5.5	7.0	89.8 ± 2.8	3.1
Cynaroside	20	94.9 ± 11.1	11.7	101.7 ± 2.6	2.6
	200	73.8 ± 7.6	10.3	109.3 ± 8.4	7.7
	4000	85.8 ± 3.0	3.5	97.6 ± 1.9	1.9
Luteolin 7-O-glucuronide	10	76.5 ± 8.3	10.8	109.1 ± 2.6	2.4
	100	69.2 ± 2.0	2.9	100.4 ± 1.2	1.2
	2000	57.4 ± 4.7	8.2	101.8 ± 6.3	6.2
Isochlorogenic acid C	5	77.3 ± 11.2	14.5	95.4 ± 13.4	14.0
	50	72.7 ± 4.4	6.1	106.4 ± 6.1	5.7
	1000	75.6 ± 3.2	4.2	90.6 ± 4.6	5.1
Cryptochlorogenic acid	5	69.8 ± 9.5	13.6	95.5 ± 3.2	3.4
	50	61.2 ± 4.1	6.7	107.5 ± 8.4	7.8
	1000	75.3 ± 1.1	1.5	109.3 ± 3.1	2.6
Chlorogenic acid	5	74.3 ± 4.1	5.5	86.6 ± 4.3	5.0
	50	84.2 ± 7.6	9.0	82.0 ± 11.5	14.0
	1000	69.6 ± 4.4	6.3	81.1 ± 2.8	3.5
Apigenin	5	80.9 ± 4.9	6.1	99.7 ± 6.1	6.1
	50	95.0 ± 4.5	4.7	71.6 ± 8.1	11.3
	1000	74.3 ± 5.6	7.5	87.2 ± 2.3	2.6
Acacetin	5	83.3 ± 8.4	10.1	104.0 ± 4.5	4.3
	50	74.8 ± 6.6	8.8	111.6 ± 10.1	9.1
	1000	78.0 ± 5.6	7.2	85.9 ± 1.6	1.9
Hyperoside	5	63.4 ± 4.5	7.1	101.3 ± 4.9	4.8
	50	70.7 ± 8.8	12.4	81.0 ± 10.7	13.2
	1000	67.6 ± 4.2	6.2	85.0 ± 2.4	2.8
Isoquercitrin	5	58.5 ± 4.3	7.4	97.2 ± 3.4	3.5
	50	56.9 ± 1.7	3.0	92.7 ± 9.9	10.7
	1000	67.6 ± 4.1	6.1	85.0 ± 2.6	3.1
Apigenin 7-glucoside	5	86.7 ± 7.6	8.8	104.4 ± 4.4	4.2
	50	81.5 ± 9.4	11.5	91.4 ± 7.0	7.7
	1000	74.9 ± 5.6	7.5	85.4 ± 3.0	3.5
Tilianin	1	75.3 ± 10.8	14.3	104.1 ± 3.3	3.2
	10	72.2 ± 7.4	10.2	100.9 ± 11.8	11.7
	200	84.6 ± 3.9	4.6	95.6 ± 1.7	1.8
Hesperidin	1	86.2 ± 11.2	13.0	103.1 ± 9.6	9.3
	10	84.5 ± 6.1	7.2	89.3 ± 10.4	11.6
	200	75.3 ± 5.6	7.4	84.6 ± 1.4	1.7

**Table 5:** Stability of 13 analytes in rat plasma (*n* = 6).

Compounds	Spiked concentration (ng/mL)	Room temperature for 6 h		Three freeze-thaw cycles		Autosampler for 12 h		-80°C for 14 days	
		Measured concentration (ng/mL)	RSD (%)	Measured concentration (ng/mL)	RSD (%)	Measured concentration (ng/mL)	RSD (%)	Measured concentration (ng/mL)	RSD (%)
Luteolin	20	20.3 ± 0.3	1.5	23.2 ± 0.7	3.0	21.3 ± 0.4	1.9	17.4 ± 0.3	1.7
	200	193.5 ± 3.3	1.7	223.7 ± 1.5	0.7	181.7 ± 0.8	0.4	195.1 ± 2.6	1.3
	4000	4419.0 ± 9.2	0.2	4475.1 ± 11.8	0.3	3656.3 ± 33.7	0.9	4195.2 ± 15.1	0.4
Cynaroside	20	20.6 ± 0.9	4.4	22.6 ± 1.0	4.4	20.0 ± 0.2	1.0	21.1 ± 0.8	3.8
	200	182.4 ± 0.6	0.3	219.8 ± 1.0	0.5	203.4 ± 6.1	3.0	205.0 ± 4.0	2.0
	4000	4360.1 ± 32.4	0.7	4152.9 ± 34.1	0.8	4158.6 ± 15.3	0.4	4060.3 ± 121.9	3.0
Luteolin 7-O-glucuronide	10	10.9 ± 0.8	7.3	11.1 ± 0.1	0.9	9.6 ± 0.3	3.1	8.8 ± 0.2	2.3
	100	110.9 ± 1.2	1.1	112.6 ± 0.5	0.4	90.7 ± 0.4	0.4	99.7 ± 0.3	0.3
	2000	2214.4 ± 17.9	0.8	2225.7 ± 9.3	0.4	1960.1 ± 38.5	2.0	1930.7 ± 22.5	1.2
Isochlorogenic acid C	5	5.2 ± 0.2	3.8	5.9 ± 0.1	1.7	4.6 ± 0.4	8.7	5.6 ± 0.1	1.8
	50	48.1 ± 0.8	1.7	55.3 ± 0.4	0.7	45.1 ± 0.6	1.3	43.3 ± 0.3	0.7
	1000	1049.2 ± 32.0	3.0	1052.4 ± 38.4	3.6	1047.3 ± 12.4	1.2	1080.3 ± 37.8	3.5
Cryptochlorogenic acid	5	5.3 ± 0.1	1.9	5.9 ± 0.1	1.7	5.0 ± 0.2	4.0	5.2 ± 0.2	3.8
	50	49.1 ± 1.5	3.1	54.1 ± 0.3	0.6	44.5 ± 1.3	2.9	46.7 ± 1.4	3.0
	1000	1012.9 ± 11.1	1.1	1122.8 ± 6.8	0.6	1069.3 ± 16.2	1.5	1075.7 ± 52.9	4.9
Chlorogenic acid	5	5.8 ± 0.1	1.7	5.8 ± 0.1	1.7	5.1 ± 0.1	2.0	5.2 ± 0.1	1.9
	50	49.7 ± 2.3	4.6	54.7 ± 0.4	0.7	43.5 ± 0.7	1.6	44.4 ± 0.3	0.7
	1000	1079.2 ± 8.3	0.8	1123.5 ± 7.3	0.6	1084.8 ± 13.6	1.3	1117.1 ± 2.0	0.2
Apigenin	5	4.9 ± 0.1	2.0	5.7 ± 0.1	1.8	5.4 ± 0.2	3.7	5.2 ± 0.1	1.9
	50	49.3 ± 1.1	2.2	50.5 ± 0.4	0.8	45.8 ± 1.3	2.8	49.3 ± 1.9	3.9
	1000	1112.4 ± 11.7	1.1	1086.4 ± 17.6	1.6	987.0 ± 11.4	1.2	1063.8 ± 62.8	5.9
Acacetin	5	4.6 ± 0.1	2.2	5.8 ± 0.1	1.7	5.3 ± 0.1	1.9	4.5 ± 0.1	2.2
	50	50.3 ± 0.8	1.6	50.2 ± 5.7	11.4	47.8 ± 0.7	1.5	50.5 ± 0.7	1.4
	1000	1126.2 ± 9.5	0.8	1032.7 ± 5.6	0.5	1030.3 ± 4.9	0.5	977.4 ± 57.9	5.9
Hyperoside	5	4.4 ± 0.1	2.3	5.7 ± 0.1	1.8	3.9 ± 0.1	2.6	4.8 ± 0.2	4.2
	50	48.7 ± 1.9	3.9	54.5 ± 2.1	3.9	41.8 ± 1.4	3.3	50.3 ± 1.8	3.6
	1000	1009.6 + 19.9	2.0	1110.2 ± 4.5	0.4	803.4 ± 2.5	0.3	997.8 ± 19.4	1.9
Isoquercitrin	5	4.7 ± 0.2	4.3	5.6 ± 0.1	1.8	4.0 ± 0.2	5.0	4.5 ± 0.1	2.2
	50	49.0 ± 1.2	2.4	54.5 ± 0.8	1.5	41.6 ± 0.5	1.2	50.7 ± 2.1	4.1
	1000	1039.7 ± 20.6	2.0	1110.5 ± 21.8	2.0	1039.7 ± 20.6	2.0	1006.3 ± 30.5	3.0
Apigenin 7-glucoside	5	5.9 ± 0.1	1.7	5.6 ± 0.4	7.1	4.3 ± 0.1	2.3	5.1 ± 0.2	3.9
	50	50.5 ± 1.0	2.0	53.7 ± 0.6	1.1	44.5 ± 0.7	1.6	51.1 ± 0.4	0.8
	1000	1086.7 ± 12.9	1.2	1112.8 ± 7.2	0.6	1077.6 ± 9.6	0.9	1108.3 ± 25.6	2.3
Tilianin	1	1.1 ± 0.1	9.1	1.0 ± 0.1	10.0	0.9 ± 0.1	11.1	0.9 ± 0.1	11.1
	10	9.9 ± 0.3	3.0	11.3 ± 0.2	1.8	9.3 ± 0.1	1.1	10.2 ± 0.2	2.0
	200	226.4 ± 0.2	0.1	209.6 ± 0.3	0.1	179.0 ± 1.2	0.7	188.6 ± 0.9	0.5
Hesperidin	1	1.1 ± 0.1	9.1	1.1 ± 0.1	9.1	1.0 ± 0.1	10.0	1.1 ± 0.1	9.1
	10	10.2 ± 0.4	3.9	10.0 ± 0.1	1.0	8.8 ± 0.1	1.1	9.5 ± 0.2	2.1
	200	213.7 ± 2.1	1.0	218.3 ± 0.7	0.3	222.7 ± 2.0	0.9	209.5 ± 2.3	1.1

**Table 6:** Pharmacokinetic parameters of 8 analytes after oral administration of *Flos Chrysanthemi* extract (*n* = 6).

Compounds	*T*_max_ (h)	*C*_max_ (ng/mL)	*T*_1/2_ (h)	Ke (1/h)	AUC_(0 − tn)_ (h·ng/mL)	AUC_(0 − ∞)_ (h·ng/mL)
Chlorogenic acid	0.25 ± 0.00	194.41 ± 56.36	0.20 ± 0.17	4.03 ± 0.90	425.83 ± 139.21	494.42 ± 204.82
Cryptochlorogenic acid	0.61 ± 0.33	89.36 ± 45.31	6.90 ± 3.40	0.12 ± 0.04	318.79 ± 97.33	431.13 ± 196.93
Cynaroside	6.38 ± 3.80	2547.84 ± 1121.18	5.01 ± 4.44	0.28 ± 0.24	19064.87 ± 19942.21	19092.57 ± 19960.87
Luteolin 7-O-glucuronide	0.50 ± 0.27	749.25 ± 195.04	6.17 ± 1.43	0.12 ± 0.03	6448.10 ± 1807.31	6731.89 ± 2276.56
Apigenin 7-glucoside	0.69 ± 0.49	96.43 ± 24.75	13.87 ± 10.15	0.25 ± 0.20	1321.88 ± 488.77	1473.6352 ± 614.27
Isochlorogenic acid C	0.40 ± 0.31	101.23 ± 73.33	0.24 ± 0.22	4.69 ± 2.04	309.13 ± 148.61	404.53 ± 228.04
Apigenin	0.50 ± 0.00	301.81 ± 87.13	11.11 ± 7.10	0.09 ± 0.05	2903.87 ± 251.44	3088.95 ± 447.45
Luteolin	0.50 ± 0.00	2079.55 ± 307.09	8.89 ± 4.01	0.10 ± 0.05	24237.94 ± 2113.55	25094.35 ± 2232.12

## Data Availability

The data used to support the findings of this study are available from the corresponding authors upon request.
